# Supplementary figures and images for: 3D imaging of undissected optically cleared Anopheles stephensi mosquitoes and midguts infected with Plasmodium parasites
Source: PLoS One. 2020 Sep 16;15(9):e0238134. doi: 10.1371/journal.pone.0238134 (PMC7494115; doi:10.1371/journal.pone.0238134)

**Figure S1**

**A**

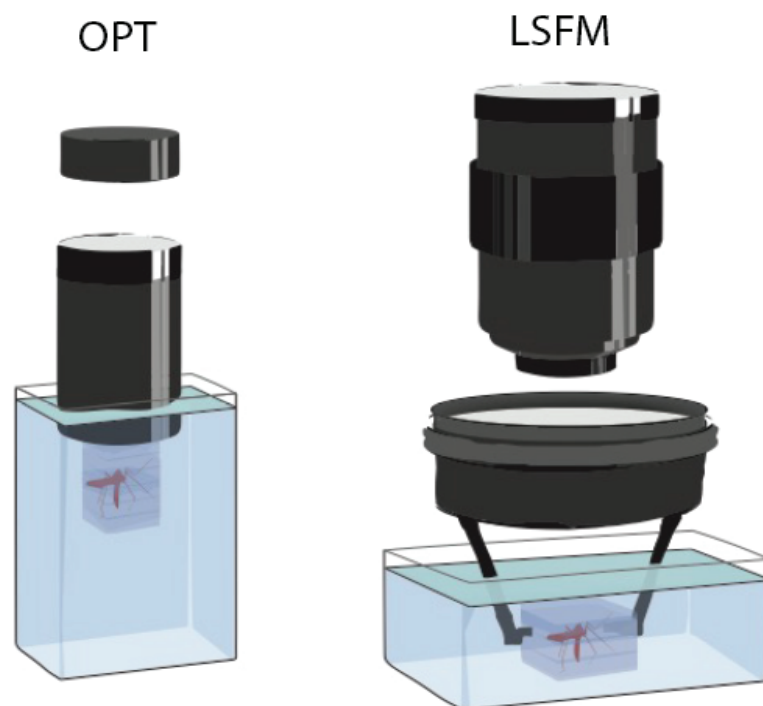

**B**

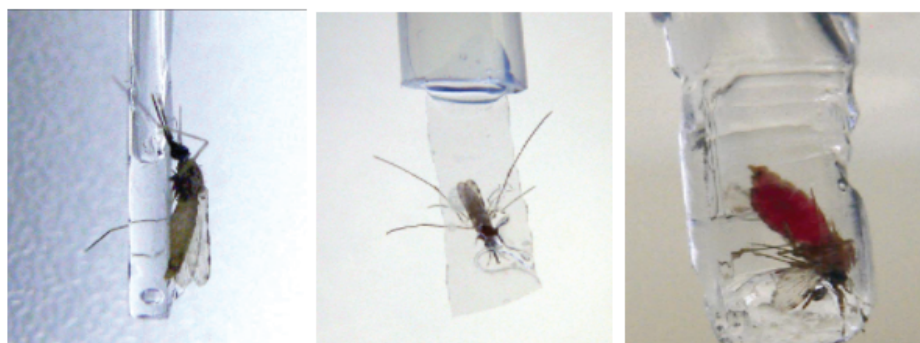

**C**

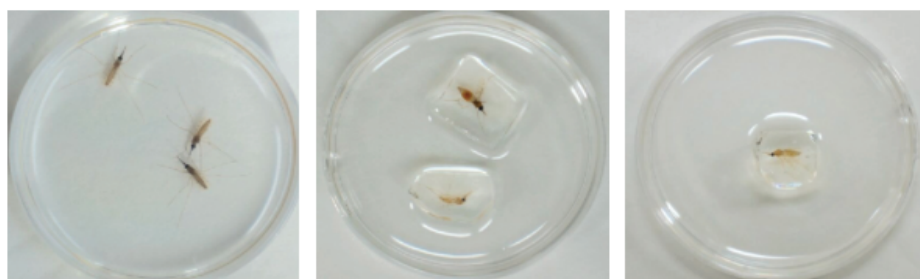

Supplement: S1 Fig — A) OPT imaging requires embedding the mosquito in low-melting temperature ultrapure agarose gel, and mounting it onto a metallic cylinder that is attached to a rotating stage via a magnet. The embedded attached mosquito is then lowered into a chamber containing index-matching liquid, such as Murray’s clear medium. The setup for Ultramicroscopy imaging involves embedding the mosquito in low-melting temperature ultrapure agarose gel, and mounting it on a lower ring of the customized holder. Both the holder and the embedded mosquito are submerged into a chamber containing index-matching liquid. B) Methods for mounting mosquitoes to enable imaging and rotation. C) Petri dishes showing (1) fixed mosquitoes prior to optical clearance and embedding and (2) optically cleared mosquitoes embedded in ultrapure low-melting temperature agarose. S1A Fig was created using BioRender.com. (PDF) [file pone.0238134.s001.pdf]

**Figure S2**

**A**

Midgut and  
blood meal

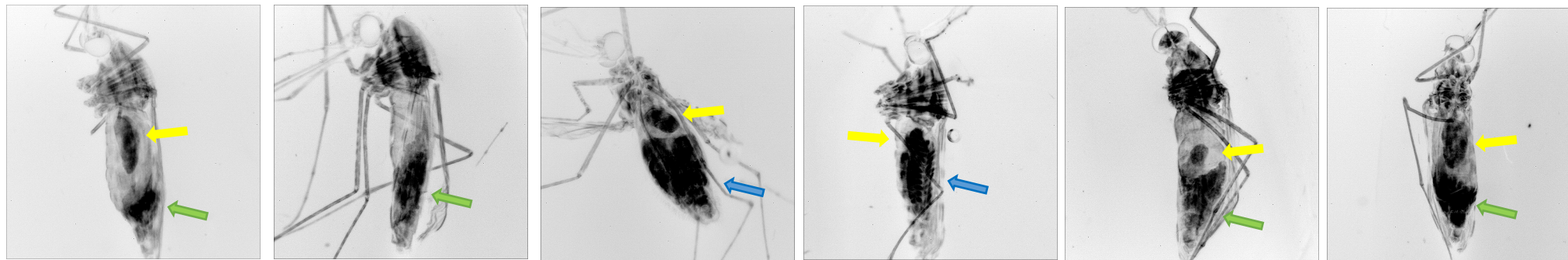

**B**

Eggs

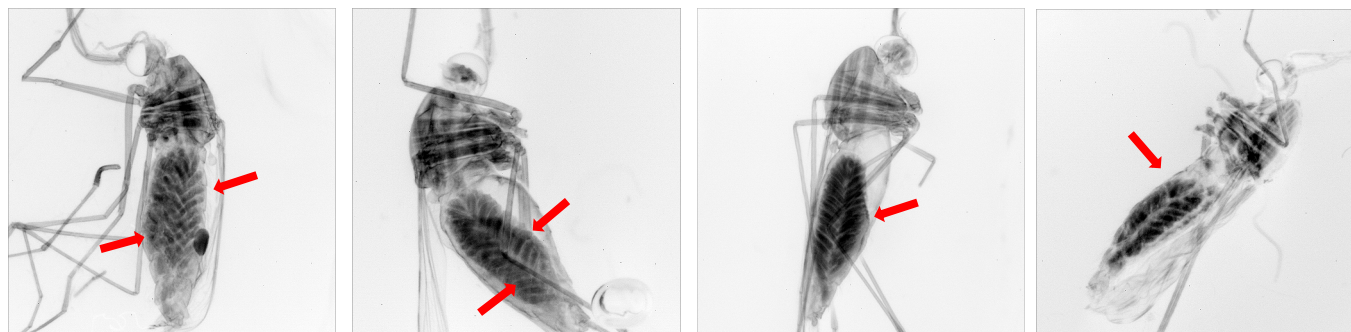

**C**

Undistinguishable  
abdominal signal

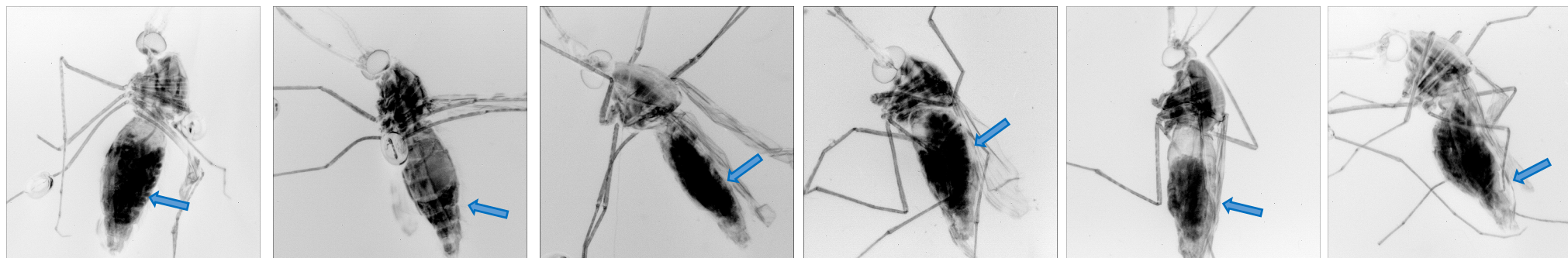

Supplement: S2 Fig — Given the very successful clearance obtained with BABB, fluorescence quenching occurs. We show in this panel various possible outcomes of clearance using BABB, including A) a mixture of detectable fluorescence in the midgut (yellow arrows), clear autofluorescence arising lower in the body (green arrows) and autofluorescence arising from eggs (blue arrows); B) clear autofluorescence arising from the eggs, but no other detectable signal in the abdomen; C) indistinguishable abdominal signal, without the possibility of distinguishing the bloodmeal from the eggs and potential parasites in the midgut. All figures were generated by the authors of this manuscript. (PDF) [file pone.0238134.s002.pdf]

**Figure S3**

Autofluorescence

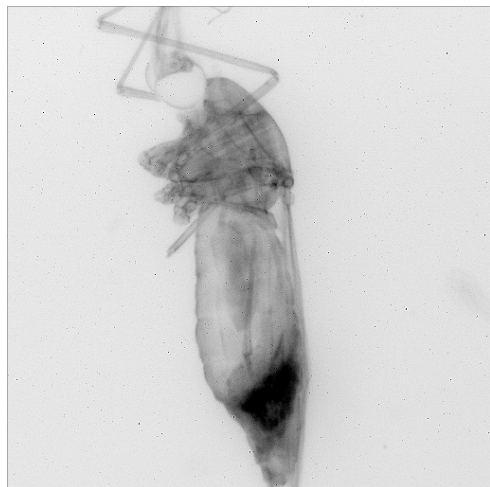

mCherry

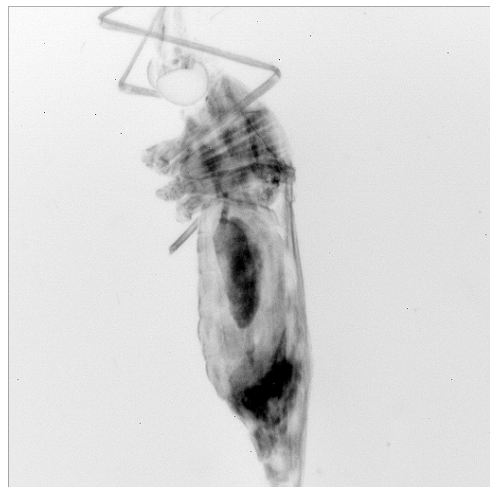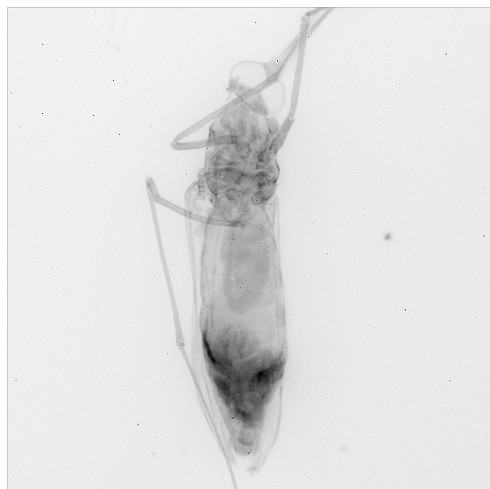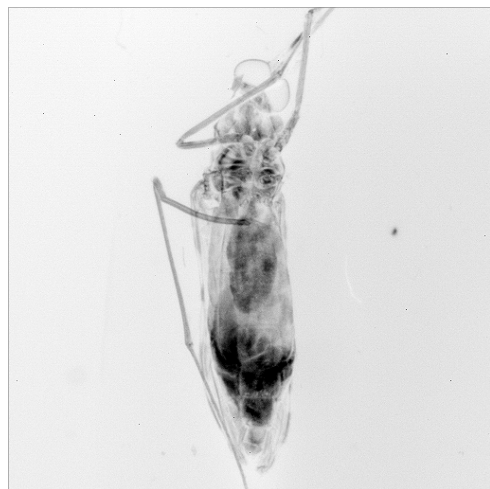

Supplement: S3 Fig — Examples obtained from S2 Fig, showing separate autofluorescence and mCherry signal, demonstrating preservation of mCherry. All figures were generated by the authors of this manuscript. (PDF) [file pone.0238134.s003.pdf]
