# Supplementary material for: 3D imaging of undissected optically cleared Anopheles stephensi mosquitoes and midguts infected with Plasmodium parasites
Source: PLoS One. 2020 Sep 16;15(9):e0238134. doi: 10.1371/journal.pone.0238134 (PMC7494115; doi:10.1371/journal.pone.0238134)
Supplement: Attachment — Submitted filename: Point to point response.docx [file pone.0238134.s006.docx]

**Point-to-point answer to reviewers**

We thank both reviewers for their helpful comments and suggestions to improve the manuscript. We reply to each comment below.

Review Comments to the Author

Reviewer #1: Some of the major problems of preparing mosquitos for microscopy are related to the cuticle’s biological properties, including the thickness, light scattering, extent of cuticle sclerotization, and autofluorescence. These properties make difficult the acquisition of live or fixed images of insects in general, especially those vectors of diseases. Recently, these problems have been overcome by adapting chemical clearing protocols for to increase the transparency of Anopheles mosquito cuticle, allowing acceptable preservation of both mosquito and Plasmodium parasites. For morphological studies to bring a novel conceptual advance, few characteristics must be attended, including good preservation of parasites and mosquito tissues, and the capacity to study host-parasite interactions in whole organisms. In this manuscript, De Niz et al., address some of these limitations by combining the use of chemical clearance methods with the power of Optical projection tomography (OPT) and light sheet fluorescence microscopy (LSFM) to successfully 1) obtain optically cleared Anopheles mosquitoes, 2) obtain 3D reconstruction of whole mosquitoes and 3) image Plasmodium parasites on infected midguts. Overall, the paper is well-executed, methodologically sound and well-written. Some figures can benefit from additional labeling to better guide the reader on the points emphasized by the authors (see comments). If further developed, this technology has the potential of establishing new protocols for studying host-parasite interactions.

We thank the reviewer for the positive overall assessment of our work.

Major comments:

Most of the discussion is a repeat of the results. The discussion would benefit from a better comparison, based on their results, of the benefits and advancement that this technology provides over other methods previously used to image infected and non-infected Anopheles mosquitoes.
A key question that the authors need to address in the discussion is what new information one can learn, given the amount of work that takes to process the samples through chemically clearance? It is not clear that the resolution level achieved allows a detailed study of host-parasite interactions. The distribution of oocysts in the midgut can be easily studied ex-vivo with well-established protocol that are less laborious, time-consuming and produce similar results. These should be discussed.

One point of concern is whether this protocol introduces morphological changes in the parasite? The low resolution of the images didn’t allow a proper evaluation of the issue.

We thank you for the comment and suggestions. We have now majorly revised the discussion. We eliminated the sections of the discussion that were redundant with the results. We instead focused the discussion, as suggested by the reviewer, on the comparison with other methods, focusing specifically on how OPT/LSFM is complementary to other techniques and overcomes major hurdles of other methods (eg. physical sectioning and laborious 3D rendering, sample preparation, or penetration depth). We discuss further the new information we can obtain from using this method, given its compatibility with fluorescence and the possibility to image the whole mosquito simultaneously. Moreover, we discuss the reviewer’s concern regarding whether the protocol introduces morphological changes in the parasite: we argue that while we can answer this question regarding the mosquito with OPT (and that we observed sample shrinkage using BABB), in the full mosquito we cannot answer whether morphological changes took place in the parasites. In dissected midguts it appears that visible morphological changes did not occur, however, we suggest to other scientists interested in using the method, that they include controls relevant to their specific question (for instance by imaging tissue sections in parallel to the transparentized mosquito).

Minor comments:

Line 170: “ScaleS and SeeDB were slowest to achieve optical clearance, yet fluorescence preservation with both methods was significantly higher than with either BABB or 3DISCO.”

The results describe a significant difference however neither the Figure 2B nor the statistics in the figure legend show statistical significance.

We have changed this sentence accordingly, to indicate that there is a tendency towards better fluorescence preservation. Indeed, the high variability resulted in a higher p-value.

Line 209: “OPT enabled imaging of the antennae, the mandibles and maxillae lining the alimentary canal, and the maxillary palps (Figure 3C, top panels). OPT also allowed detailed visualization of the proboscis and its structures (all structures mentioned above are marked with arrowheads). ”

The mandibles, macillae, and maxillary palps are not clearly distinguished in these images. The arrows just point to the mouth parts but there is not description in the legend about the parts referenced in the results text.

Line 216: “Moreover, the thorax harbors the dorsal blood vessel, the tracheal and dorsal tubes (or heart), the foregut, and various nerve ducts (Figure 3A-3B).”

These structures are not clear in the figure. It will be helpful if the authors include additional arrows or marks pointing at each structure referenced in the results. In addition, it is not clear to what structures the three black arrows are point to in Fig. 3A.

We have modified these sentence focusing only on what can be observed, making the point that different anatomical locations can be seen with varied levels of detail. We acknowledge that specific visualization of other structures might require specific labeling or more detailed imaging and reconstruction.

Line 241: “In excised cleared midguts, our technique allowed for the first time, full quantification of oocyst numbers throughout parasite development.”

The claim that their technology allows for the first-time full quantifications of oocysts is not accurate. Full quantification of oocyst numbers in dissected midguts has been done by several methods.

We have now changed this statement accordingly.

Movie 2: Slow down the speed of rotation to allow for better appreciation of the image.

 We have now slowed down the speed as suggested.

Reviewer #2: De Niz et al nicely described different microscope techniques and compared tissue clearing methods on Anopheles mosquito. The method comparison looked at time took to clear the mosquitoes, fluorescence preservation and morphological changes which are the key factors to look at in tissue clearing. The summary tables help to understand the comparisons. The 3D-reconstruction and surface rendering from OPT imaging were a beautiful work.

We thank the reviewer for the positive assessment of our work, and thank him/her for the suggestions below.

There are two points that I would like to point out.

Figure 2
It is not clear which microscope was used to quantify fluorescence preservation in figure legend and result section.

We now mention which microscope we used – we have included this information in the figure legend as requested.

Did you look into detail if you could see individual oocysts in any tested methods and assess the level of autofluorescence?

We explored this in Figure S2 and S3. We now expanded our discussion on this point further both in the results and in the discussion.

Figure 4
It is not clear what yellow rendering indicates in figure legend (line 715 to 716). It seems surface rendering of mCherry signaling. From figure legend, it could be both mcherry and egg development.

We now discuss this further both in the figure legend (lines 305-307) and in the discussion (lines 376-377).

Figure 4A, S2 & S3 show autofluorescence issue and it is hard to see individual oocysts in mosquito and identifying salivary gland sporozoite signals. For OPT and LSFM imaging, it is important to have distinguishable signals to identify the localization of protein and gene of interests. It would be great to discuss possibility to solve this issue in discussion. Not only further developing tissue clearing techniques but also the wavelength using for imaging, possibility to combine with immune-fluorescence staining advantage and disadvantage etc.

We thank the reviewer for these suggestions and have now included them in the discussion together with multiple points raised also by Reviewer 1 (lines 376-383).

Further applications (looking at sensory response, other pathogens in abdomen) using OPT and LSFM are well described in discussion and it is a great method to use.

One minor comments
line 241-244:
Have you quantified number of eggs in mosquito or dissected oocysts? (If so, how did you count it?)

We did not count it in our work, and as suggested, we refer to this as a possible application.

If this meant for possible application, it would be better in discussion section and discuss along with possible analysis strategies.

We now also discuss general quantification and image analysis methods in the discussion, which ensure the analysis or large datasets as the ones obtained from full body 3D imaging in bodies with multiple fluorophores.

We thank both reviewers overall, in specific for their helpful suggestions to improve the discussion section in our work. We think these suggestions have made it a stronger manuscript, and show better the purpose of our work, which is to provide the scientific community with a baseline method and proof of principle to further study a wide range of physiological questions.
